# Excess α-synuclein compromises phagocytosis in iPSC-derived macrophages

**DOI:** 10.1038/s41598-017-09362-3

**Published:** 2017-08-21

**Authors:** Walther Haenseler, Federico Zambon, Heyne Lee, Jane Vowles, Federica Rinaldi, Galbha Duggal, Henry Houlden, Katrina Gwinn, Selina Wray, Kelvin C. Luk, Richard Wade-Martins, William S. James, Sally A. Cowley

**Affiliations:** 1 James Martin Stem Cell Facility, Sir William Dunn School of Pathology, University of Oxford, South Parks Road, Oxford, OX1 3RE UK; 2 Department of Physiology, Anatomy and Genetics, University of Oxford, South Parks Road, Oxford, OX1 3QX UK; 3 Oxford Parkinson’s Disease Centre, University of Oxford, Oxford, OX3 9DS UK; 4 Nuffield Department of Clinical Neurosciences, University of Oxford, Oxford, OX3 9DS UK; 5 Department of Molecular Neuroscience, University College London Institute of Neurology, Queen Square, London, WC1N 3BG UK; 6 National Institutes of Health, National Institute of Neurological Disorders and Stroke, Bethesda, MD USA; 7 Department of Pathology and Laboratory Medicine, Center for Neurodegenerative Disease Research, University of Pennsylvania Perelman School of Medicine, Philadelphia, PA 19104 USA

## Abstract

To examine the pathogenic role of α-synuclein (αS) in Parkinson’s Disease, we have generated induced Pluripotent Stem Cell lines from early onset Parkinson’s Disease patients with *SNCA* A53T and *SNCA* Triplication mutations, and in this study have differentiated them to PSC-macrophages (pMac), which recapitulate many features of their brain-resident cousins, microglia. We show that *SNCA* Triplication pMac, but not A53T pMac, have significantly increased intracellular αS versus controls and release significantly more αS to the medium. *SNCA* Triplication pMac, but not A53T pMac, show significantly reduced phagocytosis capability and this can be phenocopied by adding monomeric αS to the cell culture medium of control pMac. Fibrillar αS is taken up by pMac by actin-rearrangement-dependent pathways, and monomeric αS by actin-independent pathways. Finally, pMac degrade αS and this can be arrested by blocking lysosomal and proteasomal pathways. Together, these results show that macrophages are capable of clearing αS, but that high levels of exogenous or endogenous αS compromise this ability, likely a vicious cycle scenario faced by microglia in Parkinson’s disease.

## Introduction

α-synuclein (αS) is a small, 14.5 kD, usually monomeric protein, that is highly expressed in neurons, where it can make up to 1 % of cytosolic protein, localising notably to presynaptic termini^1^. In Parkinson’s Disease (PD) it forms oligomers and fibrils, a major component of Lewy bodies and a hallmark of PD^2^. Overexpression or mutations in the *SNCA* gene, which encodes αS, can cause relatively early onset familial PD^3–5^. PD is characterised by loss of dopaminergic neurons in the *Substantia nigra*, therefore most studies focus on cell-autonomous pathological processes within dopaminergic neurons. However, non-neuronal cell processes likely also play a role in the progression of PD, with astrocytes and microglia being implicated through their expression of several key PD-related genes, including *GBA, LRRK2 and SNCA* ^6^.

Microglia are brain-resident macrophages, and are professional phagocytes, responsible for the homeostatic clearance of cellular debris, dying cells, incompetent synapses and aggregation-prone proteins. However, they can be provoked into a damaging, reactive state by inflammatory stimuli, triggering cytokine release (especially TNFα), potentially exacerbating neuronal damage and creating a vicious cycle of cytokine production and neuronal destruction (reviewed by refs 7 and 8). It is clearly important to examine the role of macrophages/microglia in clearing αS, and conversely, to understand whether the function of macrophages/microglia is affected by the presence of excess or mutant forms of the protein, as found in PD. Here, we examine this for the first time using human induced Pluripotent Stem Cells (iPSC) generated from PD patients with *SNCA* A53T mutation or the extremely rare *SNCA* Triplication. We differentiate the iPSC to adherent macrophages (pMac) via non-adherent macrophage precursors (pMacpre), following our previously published protocol^9^. These pMac represent yolk-sac-derived, tissue-resident macrophages^10, 11^ and therefore share the same ontogeny as microglia, which migrate into the fetal brain from the yolk-sac before the formation of the blood-brain barrier. Moreover, they can be skewed towards a microglial phenotype by co-culture with iPSC-neurons^12^. They are, therefore, a better model for microglia-related research questions than patient blood-derived monocytes, which derive from adult bone-marrow-hematopoiesis. pMac also overcome the limited availability of patient blood samples, and the complicating effects of medication and co-morbidities on immune cellular phenotypes.

We observe increased intracellular and extracellular αS in Triplication pMac, but not in A53T pMac. We report decreased phagocytosis by *SNCA* Triplication pMac, but not A53T pMac, and this decrease is phenocopied in healthy control pMac by addition of monomeric αS. pMac take up monomeric and fibrillar αS, degrading αS via lysosomal and proteasomal pathways. Macrophages therefore clear αS, but are easily intoxicated by higher than physiologically normal levels.

## Results

### *SNCA* Triplication, but not *SNCA* A53T mutation, causes elevated intracellular and extracellular αS protein levels in pMacpre and pMac

**Figure 1:**
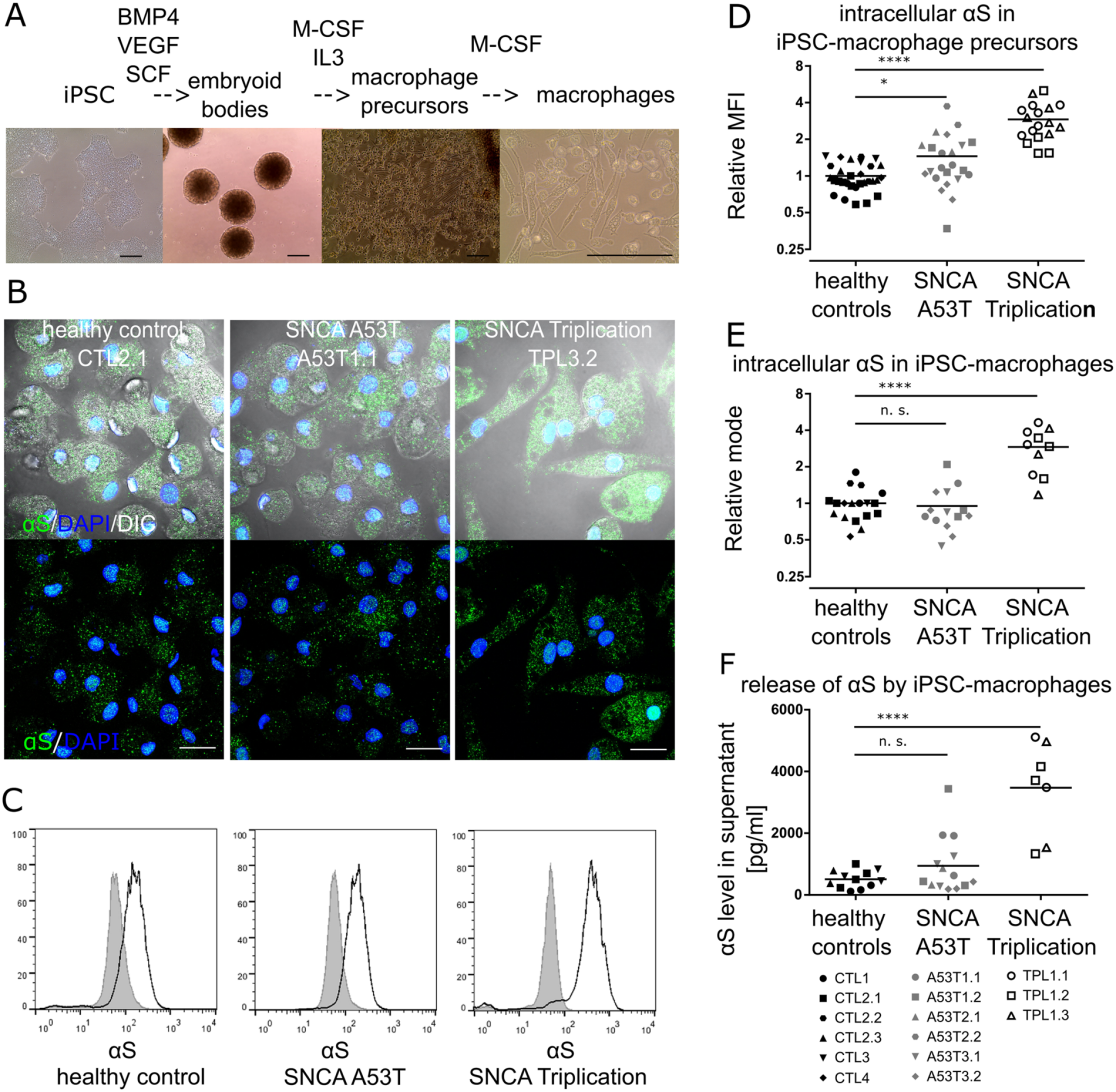
pMacpre and pMac expression of αS. (**A**) iPSC differentiation to macrophages (scale bar = 200 µm). (**B**) Intracellular staining for αS in pMac, representative z projected confocal images (scale bar = 20 µm). (**C**,**D**,**E**) Intracellular levels of αS by flow cytometry: (**C**) Representative αS FACs plots of pMacpre; (**D**) pMacpre αS levels (geometric mean fluorescence intensity, MFI); (**E**) pMac αS levels. (**F**) αS levels in 7-day supernatant from pMac. Values normalized to WT mean for each independent experiment. Also see Figs S1–3 Statistical analyses, one way ANOVA with Dunnett’s multiple comparisons test.

Multiple iPSC lines generated from 3 A53T patients, 1 Triplication patient and 4 normal control donors, all differentiated successfully to pMacpre and pMac (Table 1, Fig. 1A, Table S1, Figs S1–3). *SNCA* gene expression levels in pMac from control donors was not significantly different to levels in blood-derived monocytes and macrophages (Fig. S4). In controls and A53T pMac, αS positive puncta were found distributed throughout the cytosol by confocal microscopy, with a proportion of the signal apparently in the nucleus, as described previously for neurons^13^. Triplication pMac had many more αS puncta (Fig. 1B). Relative quantification of intracellular αS levels by flow cytometry, showed no significant difference in A53T pMacpre and pMac versus controls, but Triplication pMacpre and pMac had a significant (3-fold) increase in intracellular αS (Fig. 1C–E), and were significantly more granular as assessed by flow cytometry, whilst their size was not significantly different from controls (Figure S3B,C).

**Table 1:** iPSC lines used in this study.

ID of iPSC clone, this study	STEMBANCC/OPDC ID of iPSC clone	Diagnosis	*SNCA* Genotype	Sex	Age of Biopsy (years)	Reprogramming method	Fibroblast characterised (original ID)	iPSC clone characterised	GEO
CTL1	SFC180–01–01	healthy control	WT/WT	female	60	Cytotune1		this study	GSE89886
CTL2.1	SFC840-03-03/AH017-13	healthy control	WT/WT	female	67	Cytotune1	14	14	GSE53426
CTL2.2	SFC840-03-05							this study	GSE89886
CTL2.3	SFC840-03-06							this study	GSE89886
CTL3	SFC841-03-01	healthy control	WT/WT	male	36	Cytotune1	42	42	GSE64582
CTL4	SFC856-03-04	healthy control	WT/WT	female	78	Cytotune1		this study	GSE89886
CTL5	AH016-3	healthy control	WT/WT	male	80	rv SO³KMN	43	43	GSE77664
CTL6	SFC854-03-02	healthy control	WT/WT	male	72	Cytotune1		This study	GSE89886
A53T1.1	SFC828-03-04	PD	A53T/WT	female	51	Cytotune1	A53T_360	this study	GSE89886
A53T1.2	SFC828-03-06							this study	GSE89886
A53T2.1	SFC829-03-04	PD	A53T/WT	male	46	Cytotune1	A53T_065	this study	GSE89886
A53T2.2	SFC829-03-06							this study	GSE89886
A53T3.1	SFC830-04-08	PD	A53T/WT	male	51	Cytotune1	A53T_660	this study	GSE89886
A53T3.2	SFC830-04-09							this study	GSE89886
TPL1.1	SFC831-03-01	PD	Triplication/WT	female	55	Cytotune1	44	this study	GSE89886
TPL1.2	SFC831-03-03							this study	GSE89886
TPL1.3	SFC831-03-05							this study	GSE89886

αS levels in 7-day tissue culture medium were not significantly different for A53T pMac (1001 pg/ml ± 261; mean ± SEM, n = 13) versus control (506 pg/ml ± 81; n = 12), but were significantly higher with Triplication (3473 pg/ml ± 572; n = 7) (Fig. 1F). αS levels in XVIVO15 medium alone were at the lower detection limit of the assay (58 pg/ml ± 1.8; n = 4). These αS levels secreted by pMac were comparable to those secreted by iPSC-dopaminergic neuronal cultures (previously published^14^, measured using the same assay platform), which ranged from ~100–400 pg/ml in 2-day supernatant from controls and ~200–800 pg/ml for lines from patients harbouring N370S mutations in the Parkinson’s disease-associated gene for glucocerebrosidase, *GBA*.

**Figure 2:**
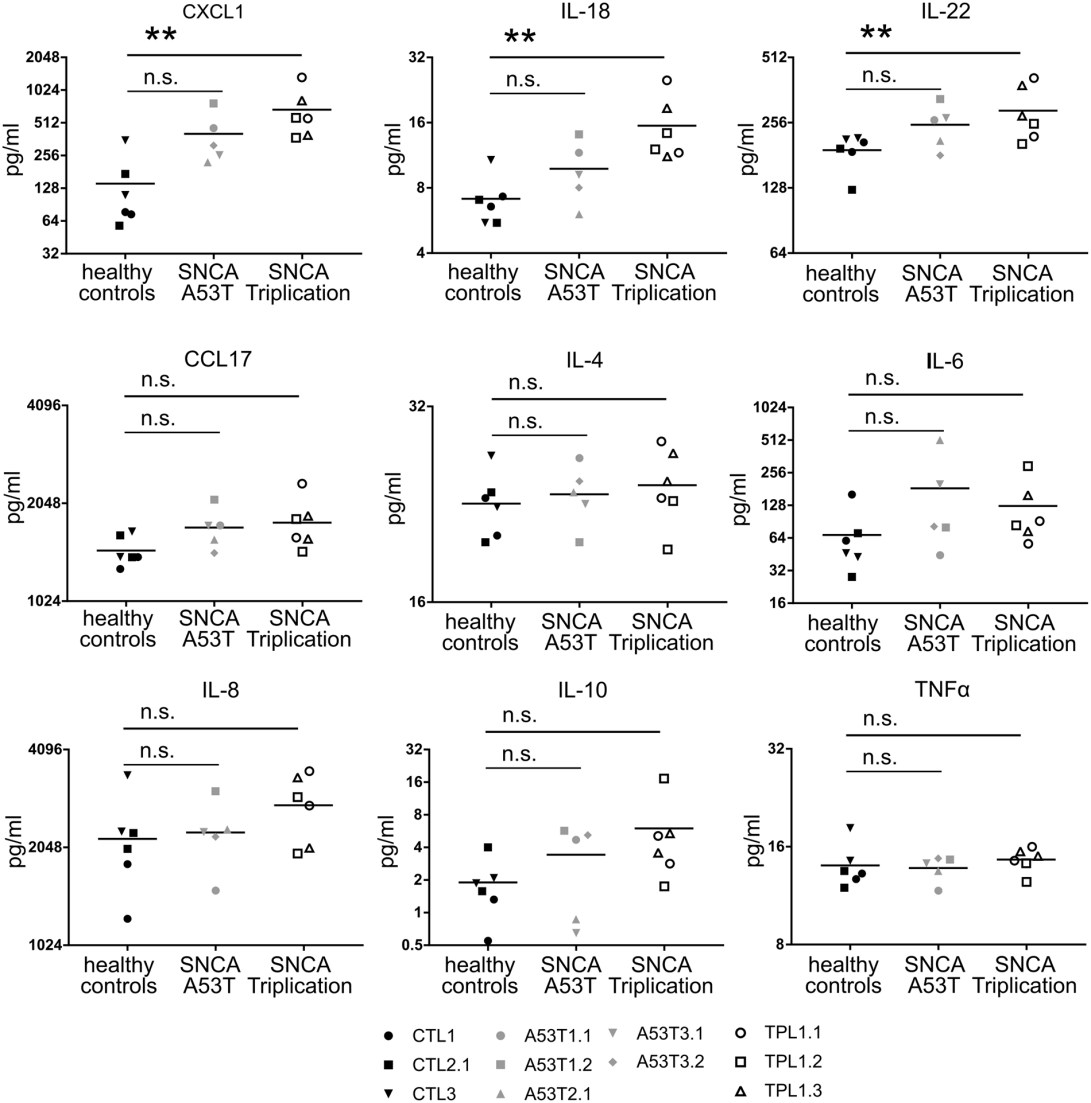
Cytokine and chemokine release by healthy control and SNCA mutant pMacs. Supernatants from pMac were assayed with Cytokine & Chemokine 34-Plex Human ProcartaPlex. Selected results are shown here, further cytokine results are shown in Table S2. Statistical analyses one way ANOVA with Dunnett’s multiple comparisons test.

Cytokines in supernatants from unstimulated pMac were not significantly different from controls for Triplication or A53T for the majority of cytokines measured, including for the key proinflammatory cytokine TNFα (34-plex, Fig. 2 and Table S2). However, the chemokine CXCL1 (GRO-α), and the proinflammatory cytokines IL-18 and IL-22 were significantly constitutively upregulated in Triplication pMac versus controls (3, 2 and 1.5-fold respectively), suggesting a modest, specific dysregulation of cytokine production in macrophages overexpressing *SNCA*.

Together, these results show that pMac express αS and release it to the medium, and that this is significantly increased in Triplication mutants, suggesting that their brain resident cousins, microglia, may contribute to accumulation of αS in the brain and possibly to the spreading of αS species.

### Excess αS levels, but not A53T mutation, reduces phagocytosis in pMac

**Figure 3:**
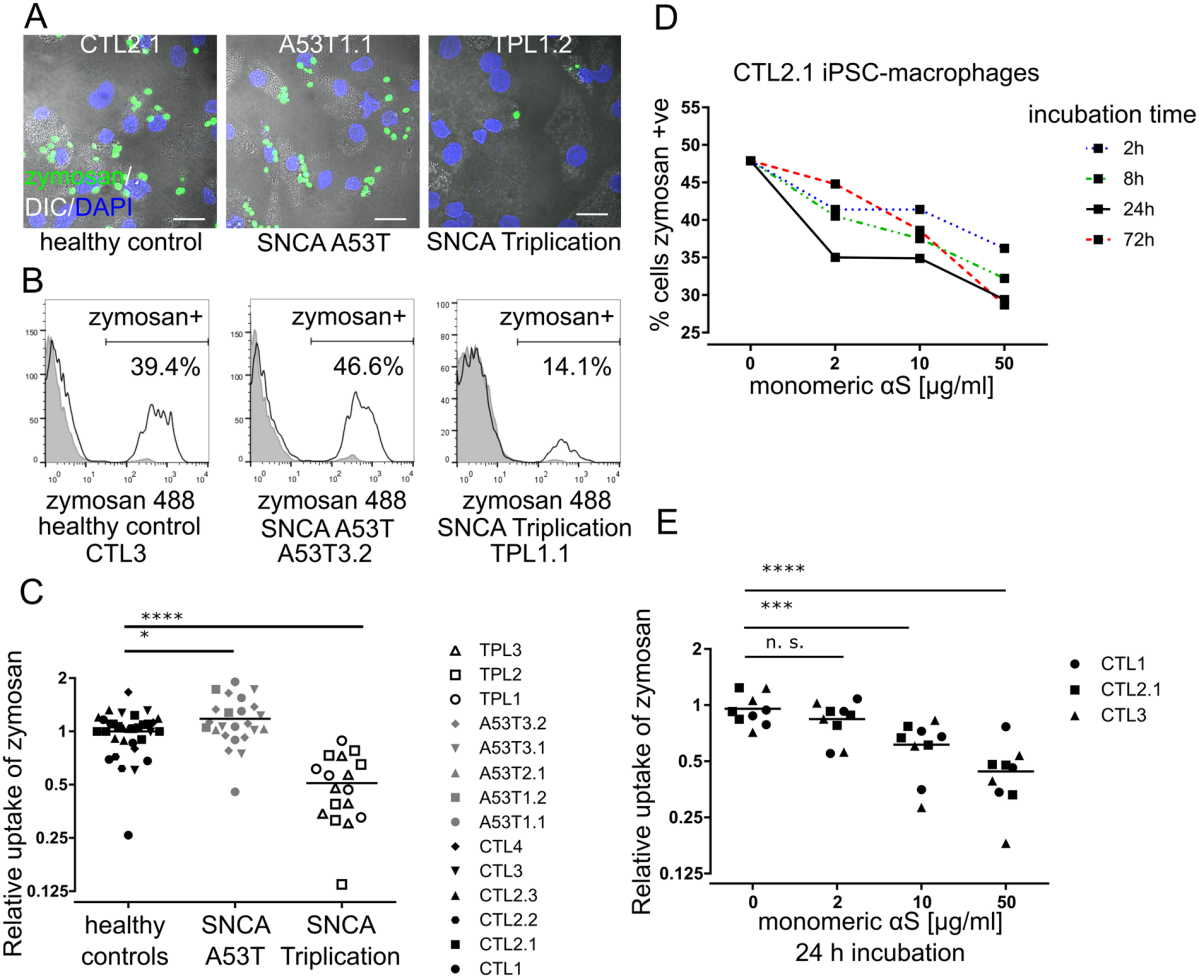
High levels of αS reduce phagocytosis in pMac. Phagocytosis was measured by uptake of fluorescent zymosan by pMac. (**A**) Z-projection of confocal images (scale bar = 20 µm). (**B**) representative FACS plots of zymosan uptake (black line, untreated with cytochalasin D; gray, cytochalasin D-treated). (**C**) Quantification of (**B**) (3 independent experiments). (**D**) Incubation with monomeric αS for different lengths of time, then challenge with zymosan (one line). (**E**) Incubation with different concentrations of monomeric αS (24 hr), then challenge with zymosan. Values normalized to untreated mean for each of 3 independent experiments (**C**,**E**). Statistical analyses, one way ANOVA with Dunnett’s multiple comparisons test. Also see Figure S4A–C.

To investigate whether phagocytosis, endocytosis or pinocytosis pathways are perturbed in αS mutant pMac, particle uptake by macrophages was assayed with Alexafluor488-conjugated killed yeast particles (zymosan)^9^, 10 kD dextran or transferrin^15^, respectively. A53T pMac had a very modest increase in zymosan uptake (118% ± 7; n = 24). Triplication lines, in contrast, showed a highly significant reduction in zymosan uptake (51% ± 4; n = 17) versus controls (n = 33) (Fig. 3A–C). Meanwhile, only very modest or no differences were detected in dextran and transferrin uptake in *SNCA* mutant lines versus controls (Figure S5A–C). Therefore, excess endogenous αS compromises phagocytic but not endocytic or pinocytic ability in pMac.

To explore whether increased levels of endogenously expressed αS was responsible for this phagocytic defect phenotype, we applied exogenous monomeric αS to the pMac. Phagocytosis of zymosan negatively correlated with monomeric αS dose and exposure time–2 µg/ml for 2 hrs showed an effect (Fig. 3D), and 10 µg/ml or 50 µg/ml for 24 hrs gave a highly significant reduction (Fig. 3E). Therefore, excess exogenous αS has the same detrimental effect as excess endogenously-derived αS on phagocytic ability of pMac, confirming that the effect seen with the Triplication lines is not due to another genetic defect in these lines.

### pMac take up fibrillar and monomeric αS

**Figure 4:**
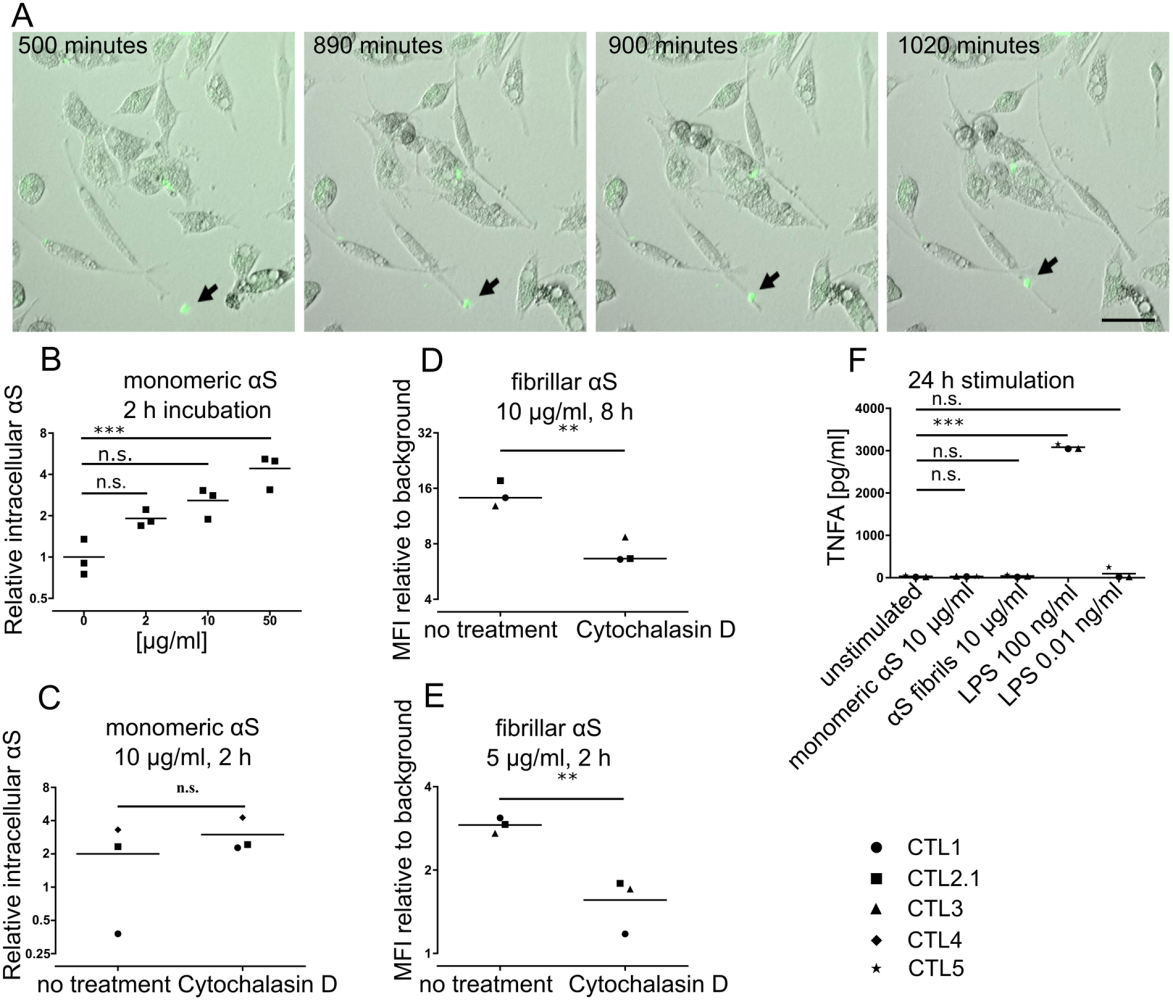
pMac take up fibrillar and monomeric αS. (**A**) 10 µg/ml Oregon Green 488-labelled fibrillar αS was added to pMac (imaged every 5 mins, 18 hrs). Frames capture one selected phagocytosis event (scale bar = 50 µm). (**B**) Intracellular αS levels following incubation with monomeric αS (FACs), normalized to mean endogenous αS levels in untreated pMac. (**C**) pMac incubated with monomeric αS, with or without Cytocholasin D (FACs as in (**B**)). (**D**) pMac incubated with fluorescent αS fibrils, with and without Cytocholasin D (FACs), MFI fluorescent αS divided by MFI medium only. (**E**) as per (**D**) but lower dose and shorter time. (**F**) TNFα levels in pMac supernatants measured 24 hrs after monomeric or fibrillar αS or LPS. Statistical analyses: (**C**,**D**,**E**) two-tailed t-test; (**B**,**F**) one way ANOVA with Dunnett’s multiple comparisons test. Also see Figure S4D–F.

We next examined whether and how αS is taken up by control pMac. Phagocytic uptake of fluorescently labelled αS fibrils was readily observable by time-lapse microscopy (Fig. 4A, Video S1). Adding exogenous monomeric αS increased intracellular levels of αS in a dose-dependent manner (Fig. 4B). 10 µg/ml for 2 hrs gave levels similar to *SNCA* Triplication, and thus, was chosen for further analyses. Uptake of monomeric αS was not affected by blocking actin polymerisation with cytochalasin D (Fig. 4C), implying uptake by mechanisms that are actin-rearrangement-independent, so not phagocytosis or macropinocytosis or actin-dependent endocytosis^16^. Further, uptake was not inhibited by Dynasore (Figure S5G), so not dynamin-dependent endocytosis, so uptake of monomeric αS could include direct traverse across the plasma membrane^17^. In contrast, uptake of fibrillar αS was significantly reduced by blocking actin polymerisation, so likely to employ phagocytosis (or possibly macropinocytosis or actin-dependent endocytosis for smaller aggregates) (Fig. 4D,E).

Lipopolysaccharide (LPS) triggered strong TNFα release in pMac (3082 ± 34; n = 3), but monomeric αS and fibrillar αS did not (29 pg/ml ± 3; n = 3; 38 pg/ml ± 14 respectively) (Fig. 4F). LPS stimulation of SNCA mutant pMac triggered the same level of TNFα release as control pMac (Supplementary Figure 5D).

Together, this shows that pMac readily take up monomeric αS by actin-rearrangement-independent pathways, and fibrillar αS by actin-dependent pathways, and are not provoked under these baseline conditions into releasing TNFα in response to αS, whether exogenous, endogenous, monomeric, fibrillar or mutant. This also shows that the phagocytic defect shown in the presence of excess αS is not the result of autocrine or paracrine TNFα signalling.

### pMac degrade αS by both lysosomal and proteasomal pathways

**Figure 5:**
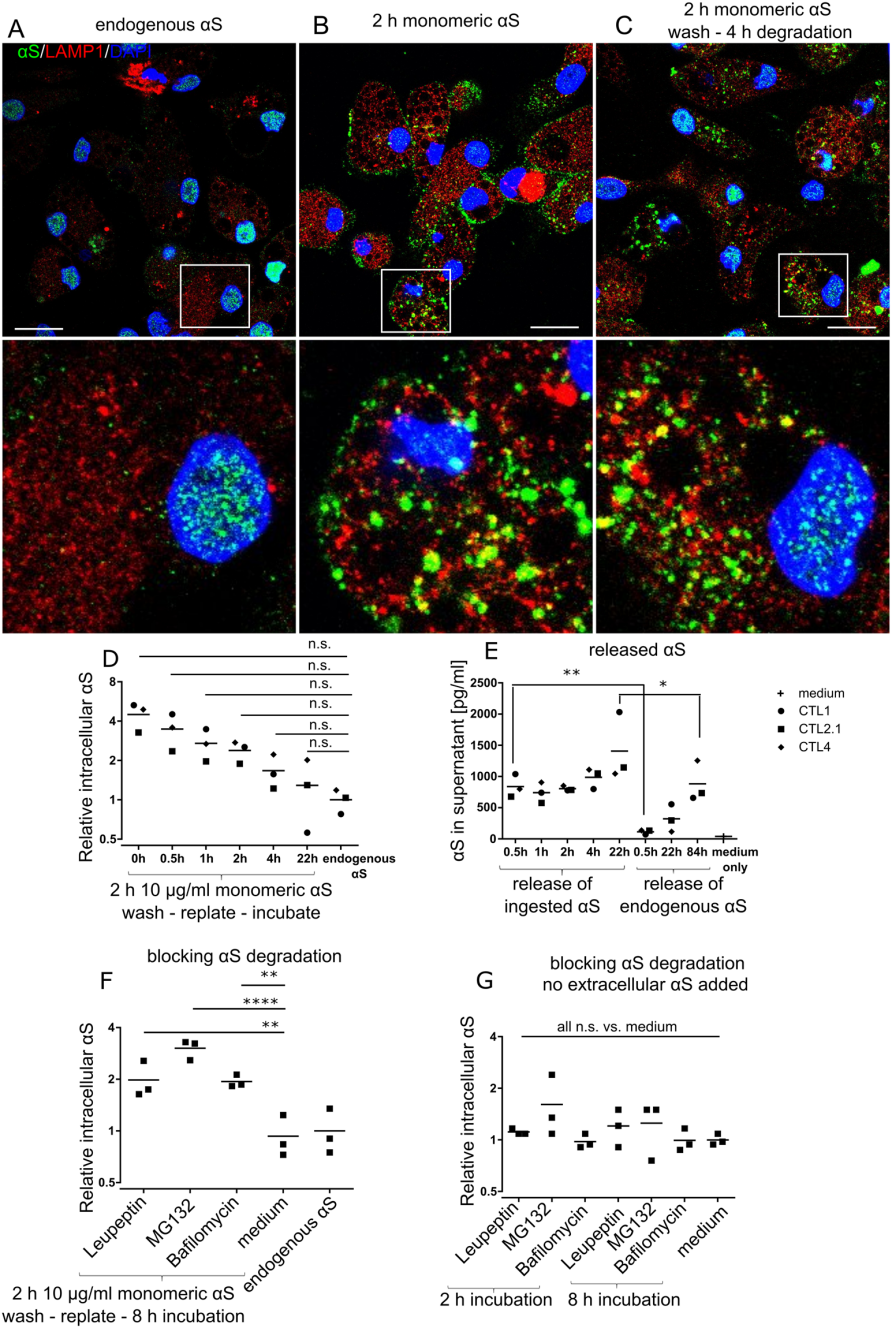
pMac degrade exogenous αS. (**A**) Immunocytochemistry of pMac: αS (green); lysosomal marker LAMP1 (red); nuclei (DAPI, blue; scale bar = 20 µm), region within white square is magnified below. (**B**) As (**A**) but pMac treated for 2 hrs with 10 µg/ml αS. (**C**) As (**B**), but pMac then washed and replated (4 hrs). (**D**) pMac incubated for 2 hrs with 10 µg/ml monomeric αS, washed and replated for the indicated number of hrs before assaying for intracellular αS by FACs; MFI relative to endogenous αS in untreated pMac. (**E**) Release of αS to supernatant of same experiment as (**D**). (**F**) As (**D**), with the addition of degradation pathway-specific drugs upon replating pMac after αS exposure and incubation for 8 hrs. (**G**) Pathway-specific drugs were added to cells (without exogenous αS exposure) for 2 and 8 hrs and assayed for intracellular accumulation of endogenous αS by FACs. (**F**,**G**) 1 line, 3 independent differentiations. Statistical analyses: (**E**) two-tailed t-test; (**D**,**F**,**G**) one way ANOVA with Dunnett’s multiple comparisons test.

To investigate whether pMac were capable of degrading exogenous αS, pMac were incubated with monomeric αS, then collected, washed and replated onto fresh plates (to completely remove any remaining extracellular αS, which readily sticks to plastic). Endogenous αS staining localised to small puncta, mainly in the nucleus, with a few puncta in the cytosol, and little co-localisation of αS with the lysosome marker LAMP1 (Figs 1A, 5A). After 2 hrs of exposure to monomeric αS, larger αS puncta were visible in the cytosol of a subset of cells, partly co-localising with LAMP1 (Fig. 5B), as well as accumulating near the cell surface. After washing, and replating for 4 hrs, αS no longer localised to the cell surface but showed increased co-localisation with LAMP1 (Fig. 5C).

The timecourse of depletion of monomeric αS from pMac was assessed by flow cytometry. αS levels in the pMac after αS-challenge and replating were ~4-fold higher than endogenous levels, indicating substantial uptake of αS, but by 22 hrs levels had dropped back to near-endogenous levels (Fig. 5D), implying that they had either expelled or degraded the excess.

To assess expulsion of αS by pMac, extracellular αS levels in supernatants from the above experiment were measured (Fig. 5E). 30 mins after replating, extracellular αS levels were similar to 4-day supernatant from untreated pMac (838 pg/ml ± 106; n = 3, and 882 pg/ml ± 188; n = 3). By 22h, levels of secreted αS nearly doubled (1407 pg/ml ± 313; n = 3), but were still about 7000 fold below input, and were below the levels measured in *SNCA* Triplication pMac supernatants.

To establish whether αS taken up by pMac is degraded within the cells, and if so by what mechanism, degradation pathway-specific drugs were added into the assay at the point of replating. Leupeptin (broad-spectrum protease inhibitor), MG132 (26S proteasome inhibitor) and Bafilomycin A1 (prevents vesicle acidification, thereby inhibiting lysosomal function) were each able to inhibit degradation of αS, as measured after 8 hrs, by 2–3-fold (Fig. 5F). Applying these pathway inhibitors to pMac not treated with exogenous αS did not significantly alter endogenous αS levels (Fig. 5G).

Together, these results show that endogenous αS is relatively stable over the timeframe of these assays, and that pMac can expel exogenously acquired αS and also degrade it by both lysosomal and proteasomal pathways.

## Discussion

This study demonstrates that αS can compromise the ability of professional phagocytes to conduct their normal homeostatic phagocytic functions, which includes clearance of αS, and likely contributes to the build-up of αS in PD patients. Our results are the first to use iPSC-derived macrophages from PD patients harbouring SNCA mutations and controls to study αS, enabling expression at the correct gene dosage in this highly relevant, authentic human cell type.

*SNCA* Triplication is extremely rare^4^. The availability of iPSC from these patients has so far been limited to samples from two individuals, a 48 year old male^18–20^ and a 55 year old female^21, 22^, the latter being the same individual from which we have derived the iPSC used in this study, and which are the first SNCA Triplication iPSC to be derived with non-integrating reprogramming vectors. Because of this rarity, we were not able to extend our results to iPSC-macrophages from additional *SNCA* Triplication patients. However, the observed phenotype was reproduced across all three clones from this patient. Moreover, we have shown for the first time that exogenous αS phenocopies the phagocytic defect displayed by *SNCA* Triplication pMac, indicating that excess αS affects phagocytosis, whether endogenous or exogenously derived.

Our results showing the negative impact of excess endogenous αS on phagocytosis concur with and extend the results of others using mice over-expressing human *SNCA* from a bacterial artificial chromosome^23^. That system relies on expression of human αS on a mouse background, and αS levels are not as precisely comparable to the *SNCA* Triplication patient as in our pMac system. Our results also concur with and extend results using *SNCA* triplication patient monocytes^23^. pMac, being entirely *in vitro*, are not exposed to any confounding or compounding *in vivo* or *ex vivo* factors, such as chronic inflammation, so results obtained with pMac likely reflect a primary defect, rather than a secondary effect. Patient monocytes may be compromised by the effects of PD drug regimens and the patients’ physiological status, whereas pMac have never been exposed to patient cytokines, drugs, etc. Consistent with this, pMac do not produce TNFα in response to monomeric or fibrillar αS or *SNCA* mutation, presumably because they have not been exposed to any priming cytokines, whereas increased TNFα shown to be released from mouse microglia overexpressing *SNCA* is likely due to chronic activation in the *in vivo* system^23, 24^.

In our system A53T SNCA did not lead to a significant increase in αS levels (Fig. 1B–E), and did not affect phagocytosis (Fig. 2A–C), in contrast to the increased αS and decreased phagocytosis seen with SNCA Triplication. This was perhaps surprising, given that in neurons A53T is associated with accumulation of αS, but our results suggest that pMac, which are professional clearers of unwanted material, are better able to process mutant, misfolded A53T αS than neurons and do not accumulate it so readily.

αS uptake by other cell types has been described previously, including free passage through the plasma membrane^17^, micropinocytosis^25^ and dynamin-dependent endocytosis^26^. Our results show for the first time that actin dynamics are involved in the uptake of fibrillar but not monomeric forms of αS in pMac, and time-lapse video of fibrillary uptake supports this. In Alzheimer’s Disease, microglia have been shown to congregate around amyloid beta plaques, yet are not able to phagocytose the plaques and instead become chronically activated^27^. We have previously shown that dopaminergic neurons harbouring PD-associated GBA mutations secrete increased levels of αS versus controls^14^. We postulate that macrophages and microglia take up αS as part of their homeostatic, non-inflammatory portfolio, but are similarly rendered less phagocytically competent by the locally very high levels of αS found in PD brains.

We explored whether pMac degrade and/or expel αS, and by what mechanisms, because if they expel substantial amounts, their microglial cousins could be potential contributors to the spread of αS in the brain. Microtubule-associated protein Tau, which forms tangles in Alzheimer’s Disease, is capable of being spread by release from murine microglia, and subsequent uptake by nearby neurons^28^. We found that pMac expelled αS both constitutively and following uptake, but lysosomal and proteasomal degradation of αS was also evident in pMac. In other cell types, αS has been shown to be ubiquitinated by Nedd4 before degradation by these routes^29, 30^. Since pMac, and by extension microglia, are capable of uptake, excretion and degradation of αS, manipulating the balance could be therapeutically useful. The use of anti-αS antibodies to promote αS uptake by microglia has already been investigated in mice^31, 32^, and humanised versions are starting to be trialled in PD patients. The pMac used here, as close cousins of microglia, offer a simple monoculture system to investigate the role of genes involved in neurodegeneration in the myeloid lineage. pMicroglia, developed from pMac recently by co-culture of pMac with iPSC-neurons by ourselves^12^ and by others along broadly similar routes^33–36^, offer an even more physiologically relevant *in vitro* system and will be used to extend the observations made here in pMac. These models will enable us to understand and be able to fine-tune the balance between successful phagocytosis/destruction of opsonised αS by microglia, and destructive microglial over-activation and/or propagation of αS species.

## Methods

Reagents were from ThermoFisher (Invitrogen) unless stated otherwise.

### Reprogramming of patient fibroblasts to iPSC and differentiation to macrophages

As previously described^9, 14^, see Supplemental Information for methodological details.

All lines were derived from dermal fibroblasts from healthy donors or Parkinson’s disease patients, through StemBANCC (SF180, SF828, SF829, SF830, SF831), or the Oxford Parkinson’s Disease Centre (SF840, SF841, SF856, SF854, AH016): participants were recruited to this study having given signed informed consent, which included derivation of hiPSC lines from skin biopsies (Ethics Committee that specifically approved this part of the study: for control donors, National Health Service, Health Research Authority, NRES Committee South Central, Berkshire, UK, REC 10/H0505/71, and for SNCA patients REC 07/H0720/161); all experiments were performed in accordance with UK guidelines and regulations and as set out in the REC.

### Determination of αS levels

pMacpre/pMac were fixed (4% PFA, 10 mins), washed (PBS), blocked/permeabilized (flow cytometry buffer: PBS, 10 µg/mL human IgG [Sigma], 1% FCS [Hyclone], 0.01% sodium azide, 0.1% Saponin [Sigma], 30 mins), incubated with rabbit anti-αS antibody (MJFR1, Abcam) or rabbit IgG1 (ab27478, Abcam) (1:250, 45 mins), washed x3 (flow cytometry buffer), incubated with donkey anti-rabbit IgG-Alexa647 (1:500), washed/resuspended (PBS) and assayed by flow cytometer (Calibur, BD) to obtain intracellular αS levels. To compare results of replicate experiments carried out on different occasions, data was normalized to the mean of healthy control lines of the corresponding experiment.

7 day supernatant from pMac cultures was centrifuged (5 mins, 400 g) to remove cell debris, transferred to a new tube and stored at −80 °C. Supernatants were applied undiluted to a human αS detection kit (MesoScale Discovery, MSD).

### Particle uptake

Uptake of dead yeast particles (zymosan) was assayed as described previously for pMac^9, 37^. Briefly, fluorescent zymosan particles were applied (2 particles/cell, 30 mins, 37 °C) in pMac differentiation medium (as a negative control for zymosan uptake, pMac were pre-treated (one hr) with 10 µM Cytochalasin D to inhibit actin polymerisation), or fluorescent dextran (D-22910) or fluorescent transferrin were applied at 50 µg/ml, fluorescence of all extracellular particles blocked (0.025% Trypan blue [Sigma] in PBS), washed (PBS), released (TrypLE Express, 10 mins), collected gently with a cell scraper, fixed (4% PFA) and analysed by flow cytometer (Calibur, BD).

### Recombinant αS

Recombinant human monomeric was purified as described previously^38, 39^. Endotoxin in αS preparations was removed using high capacity endotoxin spin columns (Pierce 88276) prior to labelling and/or aggregation into fibrils. A limulus amebocyte lysate (LAL) chromogenic kit (Pierce 88282) was used to measure endotoxin levels before and after treatment. Preparations containing endotoxin levels were >1 EU per mg of αS were used in experiments at a final dilution ≤10 µg/ml (i.e., ≤0.01 EU/ml). Monomeric αS was labeled with Oregon Green 488 succinimidyl-ester (Invitrogen) as per manufacturer’s instructions. Assembled fibrils contained a molar ratio of ≤1:10 (dye:protein). For αS uptake and degradation experiments, pMac were differentiated in ultra-low attachment surface plates (Corning) to prevent αS attachment, and to allow resuspension of pMac without the use of a cell scraper. Before and during αS stimulation pMac were treated with Cytochalasin D (Sigma, 10 µM, to inhibit actin polymerisation) or Dynasore (Cayman Chemical, 80 µM, inhibits dynamin). After αS incubation, cells were lifted by pipetting, washed 3x with PBS and either used directly for determination of αS levels or replated in fresh medium to assay αS degradation. When replating, the following drugs were used to block specific degradation pathways, as described previously^40, 41^: Leupeptin (Alfa Aesar, 100 µM, broad protease inhibitor); MG132 (Sigma, 25 µM, blocks 26S proteasome); Bafilomycin A1 (InvivoGen, 400 nM, inhibits vacuolar-type H+ ATPases, preventing vesicle acidification and lysosomal function).

### Statistical analysis

GraphPad Prism was used for statistical analysis. One way ANOVA and Dunnett’s multiple comparisons test was used for all analyses, except where indicated (for single comparisons), where Student’s 2-tailed t-test was used. n.s. = not significant, * = p < 0.05, ** = p < 0.01, *** = p < 0.001, **** = p < 0.0001. Numbers given in parentheses in the text are in the form (mean ± SEM, n).

### Accession numbers

SNP datasets and Illumina HT12v4 expression array datasets for previously unpublished iPSC lines have been deposited in GEO, under Accession number GSE89886.

## Electronic supplementary material

Supplemental information

Table S2: Cytokine profile of SNCA mutants

pMac take up fibrillar αS
