# Supplementary material for: Excess α-synuclein compromises phagocytosis in iPSC-derived macrophages
Source: Sci Rep. 2017 Aug 21;7:9003. doi: 10.1038/s41598-017-09362-3 (PMC5567139; doi:10.1038/s41598-017-09362-3)
Supplement: Supplementary file 1 — Supplemental information [file 41598_2017_9362_MOESM1_ESM.pdf]

Supplementary Information

Excess α-synuclein compromises phagocytosis in iPSC-derived macrophages

Walther Haenseler, Federico Zambon, Heyne Lee, Jane Vowles, Federica Rinaldi, Galbha Duggal, Henry Houlden, Katrina Gwinn, Selina Wray, Kelvin C. Luk, Richard Wade-Martins, William S. James, Sally A. Cowley

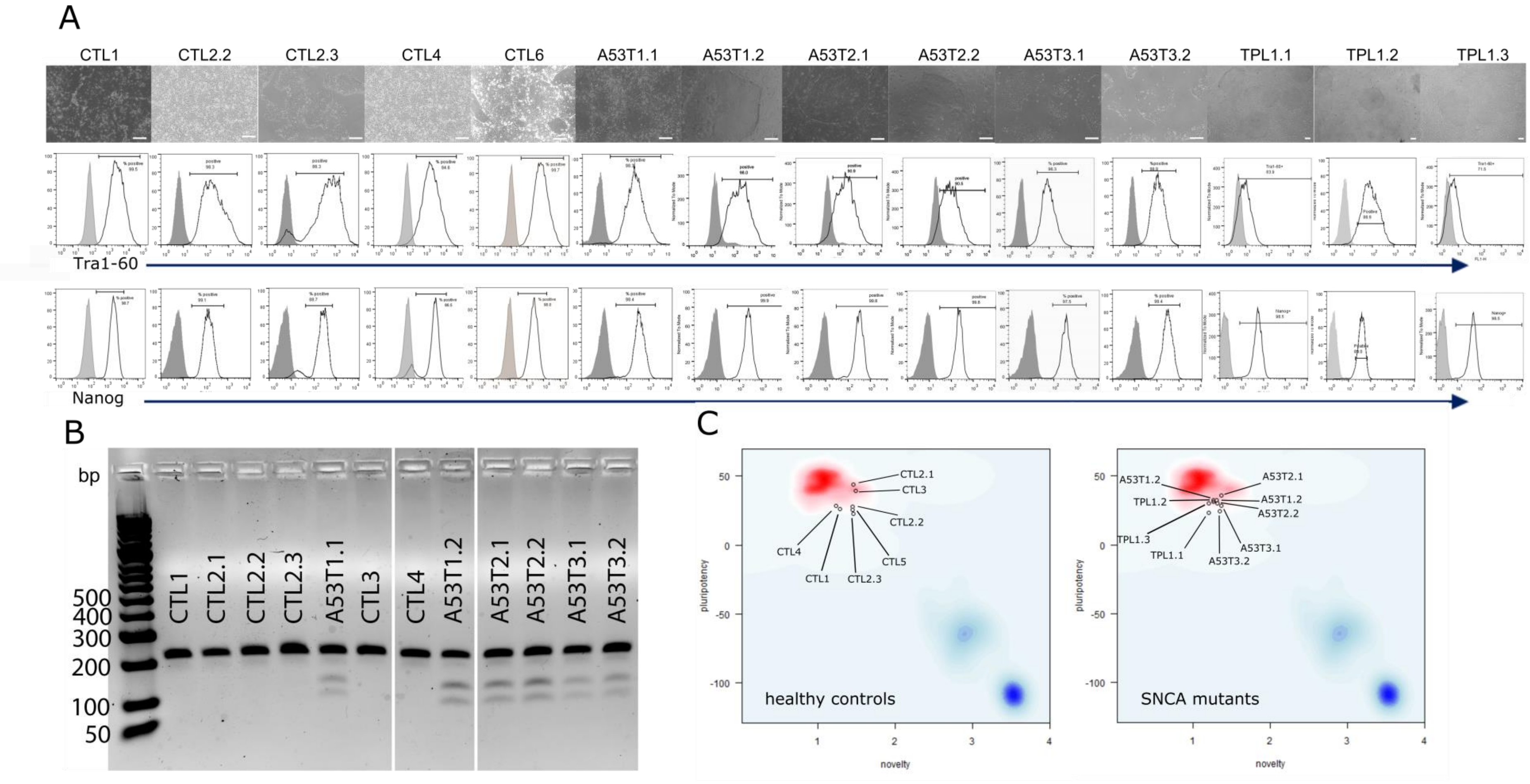

Figure S1. Characterisation of previously unpublished iPSC lines (see also Table 1)

(A) Morphology of iPSC lines and FACS for pluripotency markers Tra1-60 and NANOG, Scale bar 100 µm (B) TSP45 digest confirms heterozygous *SNCA* A53T mutation in iPSC by the presence of two extra bands of 133 bp and 88 bp (C) Pluritest confirms pluripotent expression profile, all lines clustering in the top left quadrant.

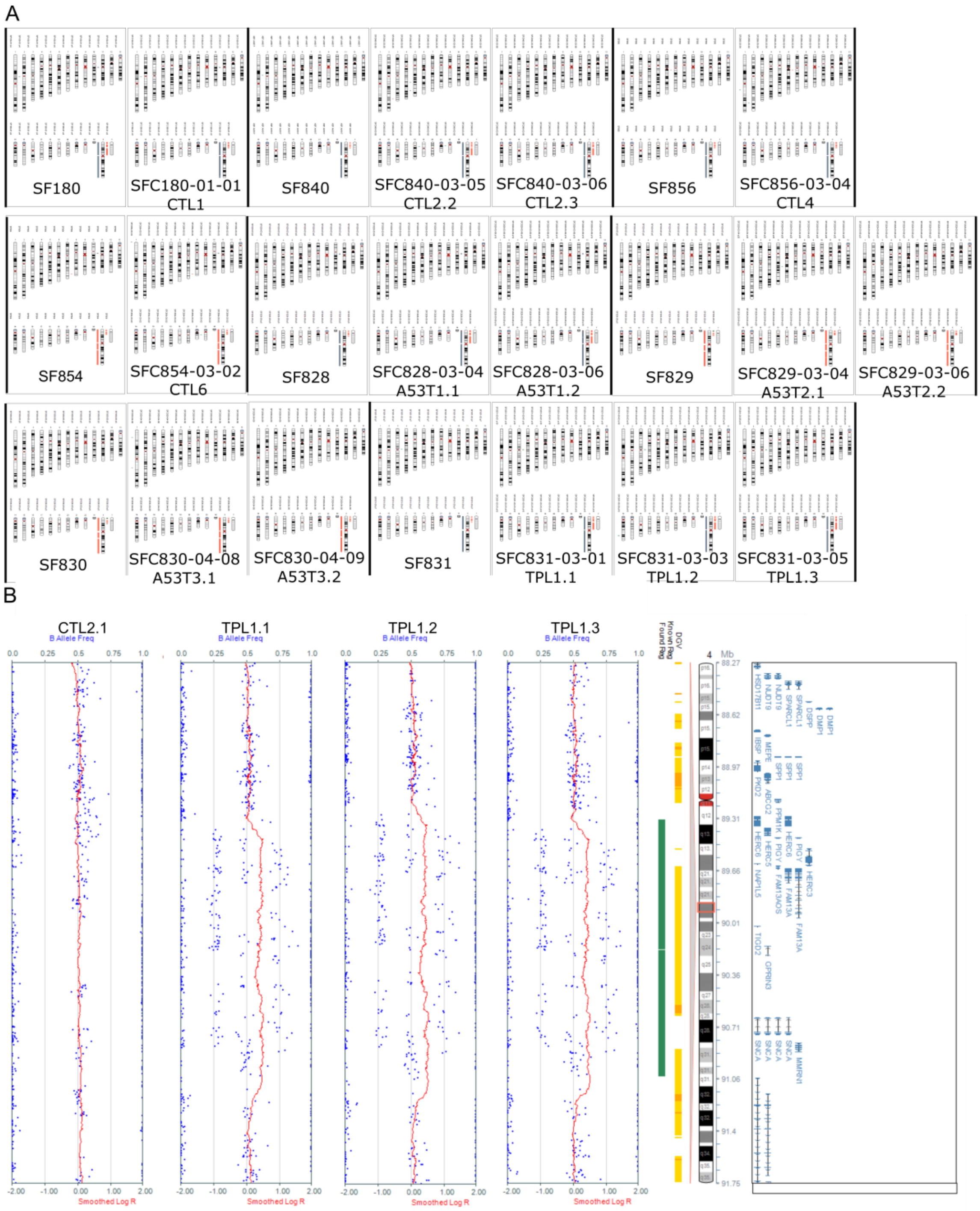

Figure S2. SNP array data (see also Table 1)

(A) Karyograms produced from SNP array show no gross abnormalities in the previously unpublished iPSC lines used in this study. Red indicates loss or single copy, green indicates gain of copy, grey indicates loss of heterozygosity on autosomes, or two copies of X chromosome (i.e. female lines). (B) SNP array confirms triplication of *SNCA* region in iPSC lines from *SNCA* triplication patient. This is as previously described on chromosome 4, position 89323214-91046719, including the genes *HERC6; HERC5; PIGY; HERC3; NAP1L5; FAM13AOS; FAM13A; TIGD2; GPRIN3; SNCA; MMRN1*.

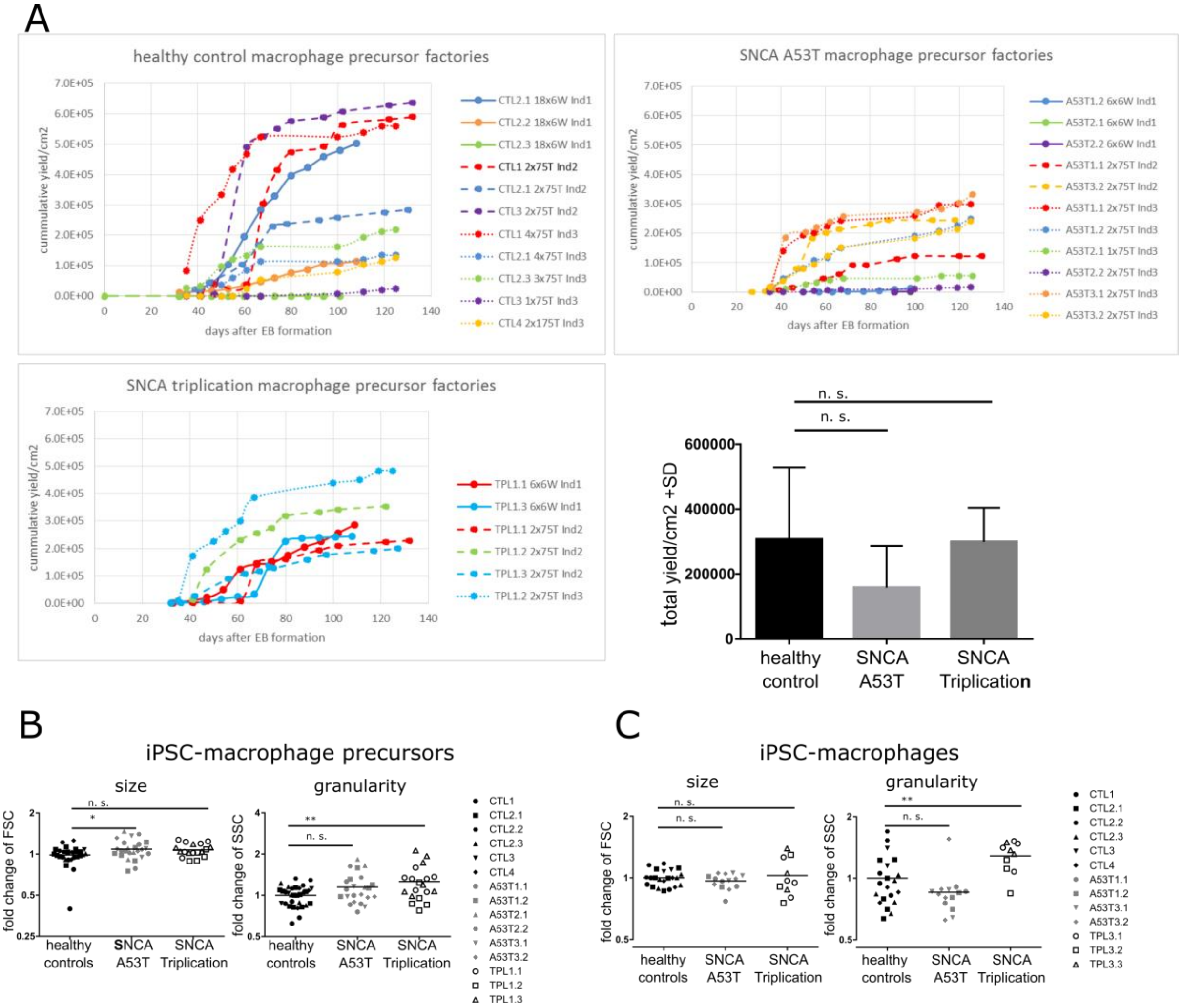

Figure S3. pMacpre yields and pMacpre/pMac morphometry (see also Table 1)

iPSC lines were differentiated to non-adherent macrophage precursors (pMacpre) and then to pMac following our previously described protocol (van Wilgenburg et al., 2013) (summarised in Figure 1A). All lines formed normal embryoid bodies, and started to release pMacpre into the supernatant after about a month. (A) Yields of 3 inductions of pMacpre factories, normalised to tissue culture plastic area, showing batch to batch variability, but no significant difference between healthy control and *SNCA* mutant lines. Differentiation culture 1 in 6 well plates, 2 in 75T flasks and 3 in 75T and 175T flasks. (B, C) Forward scatter and Side scatter flow cytometry data of αS-stained pMacpre and macrophages (from Figure 1) were analysed to compare size and granularity respectively.

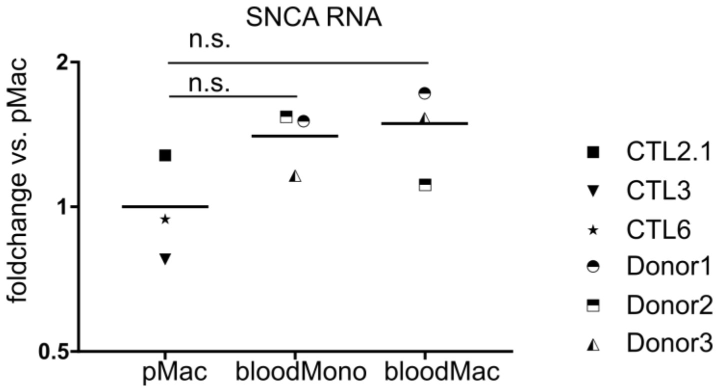

Figure S4. SNCA gene expression

SNCA RNA levels of iPSC derived macrophages (pMac) were compared to PBMC derived blood monocytes (bloodMono) and blood monocyte derived macrophages (bloodMac) of three healthy donors. Statistical analyses one way ANOVA with Dunnett's multiple comparisons test.

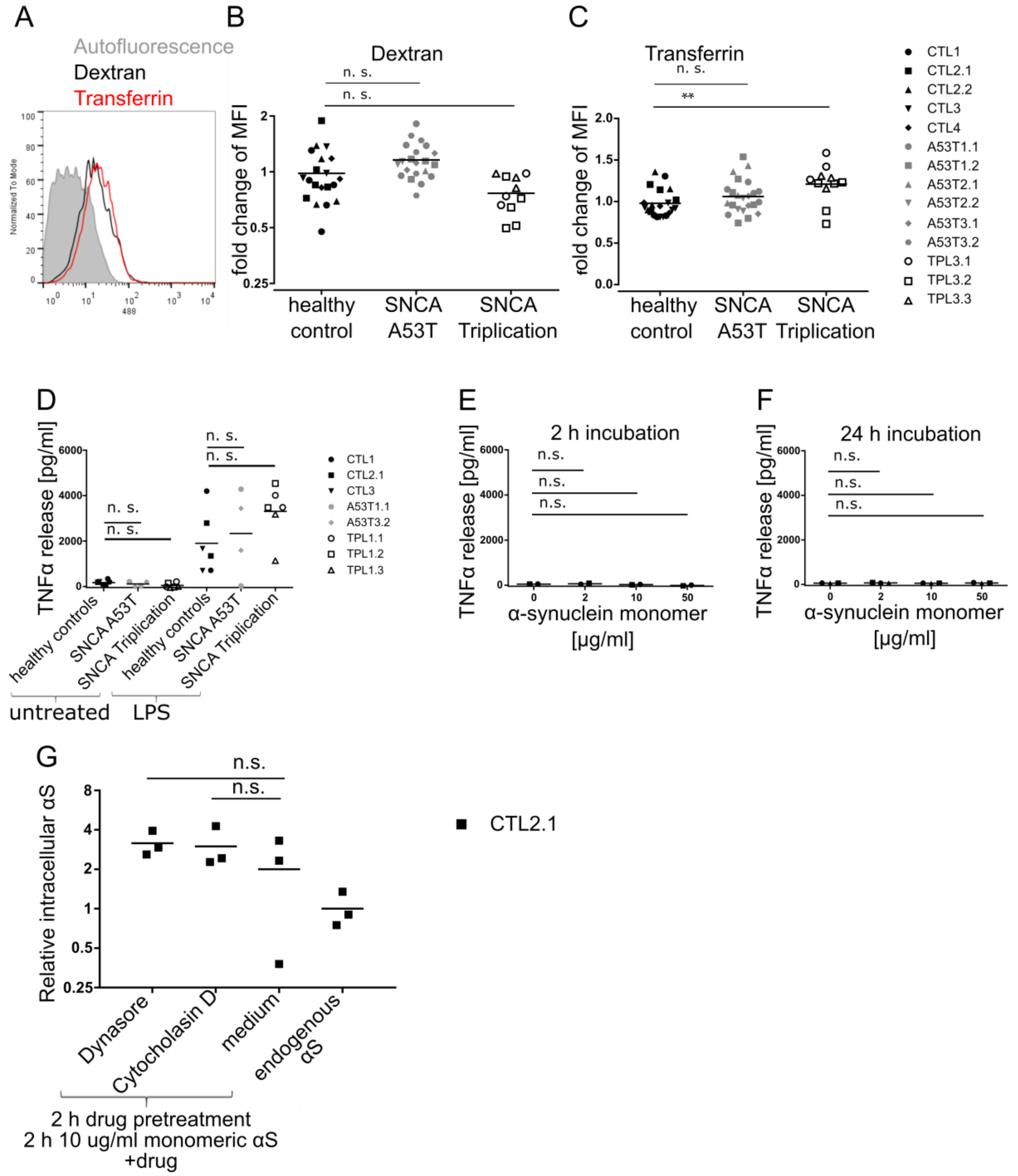

Figure S5. Further phenotypic characterisation of *SNCA* macrophages (see also Figure 2)

(A, B, C) Assessing macrophage pinocytosis and endocytosis. To assess pinocytosis macrophages were incubated with 50 µg/ml fluorescent dextran and to assess endocytosis macrophages were incubated with 50 µg/ml fluorescent transferrin or with medium only for 30 minutes. (A) Representative FACS plots. (B, C) Mean fluorescence intensity (MFI) normalised to mean of controls for each independent experiment. (D) TNFα release from macrophages from healthy controls and *SNCA* mutants, either untreated or LPS treated for 24 hours showed a strong response to LPS, but no significant difference between groups. (E) Incubation of macrophages with monomeric α-synuclein does not induce TNFα release after 2 hours, or 24 hours (F). (G) pMac were incubated with Dynasore, cytochalasin D or medium only to test the effect of these drugs on monomeric αS uptake. Statistical analyses, one way ANOVA with Dunnett's multiple comparisons test.

Table S1 Parkinson’s Patient Clinical Features (see also Table 1)

SFC828 PD onset at 39 years, severe PD, associated cognitive problems at the time of biopsy at age 51 years.
SFC829 PD onset at 40 years, mild PD with depression at the time of biopsy at 46 years.
SFC830 PD onset 42 years, moderate PD with depression at the time of biopsy at 51 years.
SFC831 AAO late 30’s, severe end stage PD, bedbound, dependant at the time of biopsy at 55 years.

Supplemental Methods

Reprogramming of patient fibroblasts to iPSC

Reagents were from ThermoFisher (Invitrogen) unless stated otherwise. Derivation of iPSC lines and QC analyses were carried out as described previously (Fernandes et al., 2016). Briefly, skin biopsies were cultured to promote outgrowth of fibroblasts, which were reprogrammed at passage 3-5, using Cytotune Sendai virus reprogramming kit (Invitrogen) according to the manufacturer's instructions (scaled down to 50,000 fibroblasts). At day 7, transduced fibroblasts were seeded onto CF1 outbred mouse embryonic feeder cells (MEF, Merck) on gelatin-coated plates (Sigma) and cultured in standard KnockOut ES medium containing 20% KnockOut Serum Replacement and 10 ng/mL bFGF (R&D), which was MEF-conditioned from day 10 onwards. Colonies displaying iPSC morphology were picked and passaged on MEFs by manual dissection, then adapted to feeder-free culture conditions in mTeSR1 (StemCell Technologies), on hESC-qualified Matrigel-coated plates (BD), passaging as clumps using 0.5 mM EDTA in PBS (Beers et al., 2012). Large-scale SNP-QCed batches were frozen at p15-25 and used for experiments within a minimal number of passages post-thaw to ensure consistency.

For FACs assessment of pluripotency markers TRA-1-60 and Nanog (B119983, IgM-488, Biolegend; 2985S, IgG-647, Cell Signaling, with appropriate isotype controls, using the same concentration and supplier), cells were fixed for 10 minutes in 2% paraformaldehyde in PBS (Alfa Aesar), permeabilised in 100% methanol at -20 degrees celsius for at least 30 mins before staining, and measurement was by FACS Calibur (Becton Dickinson), with analysis using FlowJo.

For assessment of clearance of Cytotune Sendai virus-delivered reprogramming genes, RNA was isolated using an All-Prep kit (Qiagen), reverse transcribed using a RetroScript kit (Ambion), with 2 µg template RNA in a 20 µl reaction, 2 µl of 1:10 dilution of cDNA product was used in a 25 µl RT-PCR reaction, performed according to the manufacturer's instructions and run on a 1.5% agarose gel with Log2 ladder (NEB). Positive controls (fibroblasts infected 5 days previously) were always run in parallel. Primers were SeV F: GGATCACTAGGTGATATCGAGC, R: ACCAGACAAGAGTTTAAGAGATATGTATC 181bp; SOX2 F: ATGCACCGCTACGACGTGAGCGC, R: AATGTATCGAAGGTGCTCAA 451bp; KLF4 F: TTCCTGCATGCCAGAGGAGCCC, R: AATGTATCGAAGGTGCTCAA 410bp; c-MYC F: TAACTGACTAGCAGGCTTGTCG, R: TCCACATACAGTCCTGGATGATGATG 532bp; OCT4 F: CCCGAAAGAGAAAGCGAACCAG, R: AATGTATCGAAGGTGCTCAA 483bp; β-Actin control Eurogentec 92 bp.

Genome integrity was assessed by Illumina Human CytoSNP-12v2.1 beadchip array (~300,000 markers) or OmniExpress24 array (700,000 markers), with genomic DNA made using an All-Prep kit (Qiagen) and analysis used GenomeStudio and Karyostudio software (Illumina). Ancestry plots served as a tracking QC to confirm that the iPSC lines derived from the parental fibroblasts, using a customized SNP analysis pipeline in StemDB, which hosts StemBANCC datasets (https://www.stemdb.org).

The triplication mutation was confirmed in iPSC lines derived from the triplication patient fibroblasts, by interrogating the SNP dataset using Karyostudio. The triplication region is defined as chr4:89,375,425 to 90,880,891, spanning the following genes: *HERC5*, *PIGY*, *HERC3*, *NAP1L5*, *FAM13AOS*, *FAM13A*, *TIGD2*, *GPRIN3*, *SNCA* and *MMRN1 (Devine et al., 2011)*. Assessment of conformity to pluripotent gene expression profile was performed using Pluritest (Muller et al., 2011)(pluritest.org). RNA extraction used RNeasy mini kit (Qiagen) and gene expression array used Illumina's Human-HT-12-v4 expression BeadChip according to Illumina's protocol.

Differentiation of iPSC to Macrophage precursors and Macrophages

IPSC were differentiated to macrophage precursors (pMacpre) and macrophages (pMac) as previously described (van Wilgenburg et al., 2013). In short, 4 million iPSC were seeded into an AggreWell 800 well (Stemcell Technologies) according to the manufacturer's instructions, to form EBs, in mTeSR1 and fed daily with medium supplemented with 50 ng/mL BMP-4 (Peprotech), 50 ng/mL VEGF (Peprotech) and 20 ng/mL SCF (Miltenyi Biotec). After 4 days EBs were collected and setup as macrophage factories in either 6 well plates (15 EBs/well), T75 flasks (75 EBs/well) or T175 flasks (150 EBs/well) in X-Vivo15 (Lonza), supplemented with 100 ng/mL M-CSF (Invitrogen), 25 ng/mL IL-3 (R&D), 2 mM GlutaMAX (Invitrogen), 100 U/mL penicillin and 100 µg/mL streptomycin (Invitrogen), and 0.055 mM β-mercaptoethanol (Invitrogen), fresh medium was added weekly. pMacpre emerging into the supernatant (after approximately 1 month) were collected weekly and the differentiation cultures replenished with the equivalent volume of medium. pMacpre were passed through a

40 µm cell strainer (Corning) to obtain a single cell suspension. pMac were either used directly for assays or plated onto tissue-culture treated or ultra-low attachment surface plates plastic at a standard density of 100,000 per cm^2 and differentiated for 7 days to pMac in X-Vivo15 supplemented with 100 ng/mL M-CSF, 2 mM GlutaMAX, 100 U/mL penicillin and 100 µg/mL streptomycin.

Immunostaining

pMac cultured on 8-chamber glass slides (Ibidi, 100,000 cells/cm^2) were fixed (4% PFA, 10 mins), washed (PBS), permeabilised (0.3 % TritonX-100 in PBS), blocked (normal donkey serum (Sigma), 60 mins), incubated with rabbit anti αS (MJFR1, Abcam) and mouse anti-LAMP1 (Abcam) (1:500 in PBS with 0.1% TritonX-100 and 5% normal donkey serum, overnight, 4 ^{o}C), washed x3, incubated with donkey anti-rabbit IgG Alexa647 and donkey anti-mouse IgG Alexa568 (1:500 in PBS with 0.1% TritonX-100 and 5% normal donkey serum, 90 mins, RT), washed x3 times and nuclei were stained with DAPI. Images were acquired by confocal microscopy (FV1200 Olympus) and compiled in Image J.

Preparation of cells for SNCA qPCR

iPSC-derived macrophage (pMac), were lysed directly in the well for RNA isolation. 30 ml of peripheral blood was collected from 3 healthy adult volunteers, according to University of Oxford OHS policy document 1/03, with signed informed consent. PBMCs were isolated after density gradient centrifugation with Ficol-Paque PLUS (17-1440-03, GE Healthcare), and monocytes (bloodMono) were extracted with CD14 MACS beads (130-050-201, Miltenyi). Blood monocyte-derived macrophages (bloodMac), were obtained by differentiating PBMCs on tissue-culture-treated plates for 1 week in macrophage differentiation medium. RNA was isolated with an RNeasy kit (Qiagen). RNA was reverse transcribed using High-Capacity RNA-to-cDNA Kit (Thermo Fisher). Qunatitative real time PCR was performed with *Power* SYBR Green PCR Master Mix (Thermo Fisher) on a StepOnePlus Real-Time PCR System. Primers used are SNCA_F: `GTAGCCGTGATGTGGTCATTT,` SNCA_R: `CTGTGCGCCCAGATTACCT and` endogenous control was 18S (Eurogentec)

ELISA and Multiplex cytokine array

TNFα ELISA (Ready Set Go; eBioscience) was used according to the manufacturer's instructions. Cell culture supernatants were centrifuged to pellet residual cells and debris, and the resulting cleared supernatants diluted 1:5 in assay diluent before adding to the ELISA plate. LPS (100 ng/ml; Sigma) was used as a positive control and as an activator or at 0.01 ng/ml to control for the low endotoxin levels expected in αS preparations. Cytokine & Chemokine 34-Plex Human ProcartaPlex Panel 1A (ThermosFisher) was used according to manufactures instructions. Cell culture supernatants were added undiluted and the bead were analysed with a Luminex100 system (BioRad).

Supplemental References

Beers, J., Gulbranson, D.R., George, N., Siniscalchi, L.I., Jones, J., Thomson, J.A., and Chen, G. (2012). Passaging and colony expansion of human pluripotent stem cells by enzyme-free dissociation in chemically defined culture conditions. Nature protocols *7*, 2029-2040.

Devine, M.J., Ryten, M., Vodicka, P., Thomson, A.J., Burdon, T., Houlden, H., Cavaleri, F., Nagano, M., Drummond, N.J., Taanman, J.W.*, et al.* (2011). Parkinson's disease induced pluripotent stem cells with triplication of the alpha-synuclein locus. Nature communications *2*, 440.

Fernandes, H.J., Hartfield, E.M., Christian, H.C., Emmanoulidou, E., Zheng, Y., Booth, H., Bogetofte, H., Lang, C., Ryan, B.J., Sardi, S.P.*, et al.* (2016). ER Stress and Autophagic Perturbations Lead to Elevated Extracellular alpha-Synuclein in GBA-N370S Parkinson's iPSC-Derived Dopamine Neurons. Stem cell reports *6*, 342-356.

Muller, F.J., Schuldt, B.M., Williams, R., Mason, D., Altun, G., Papapetrou, E.P., Danner, S., Goldmann, J.E., Herbst, A., Schmidt, N.O.*, et al.* (2011). A bioinformatic assay for pluripotency in human cells. Nature methods *8*, 315-317.

van Wilgenburg, B., Browne, C., Vowles, J., and Cowley, S.A. (2013). Efficient, long term production of monocyte-derived macrophages from human pluripotent stem cells under partly-defined and fully-defined conditions. PloS one *8*, e71098.
